# Supplementary material for: Causal Relationship between Aging and Anorexia Nervosa: A White-Matter-Microstructure-Mediated Mendelian Randomization Analysis
Source: Biomedicines. 2024 Aug 16;12(8):1874. doi: 10.3390/biomedicines12081874 (PMC11351342; doi:10.3390/biomedicines12081874)
Supplement: Supplementary file 1 [file biomedicines-12-01874-s001.zip › Supplementary Figure/Figure S4/Singe SNP plot of MR analysis for TL to ubm-b-1706.pdf]

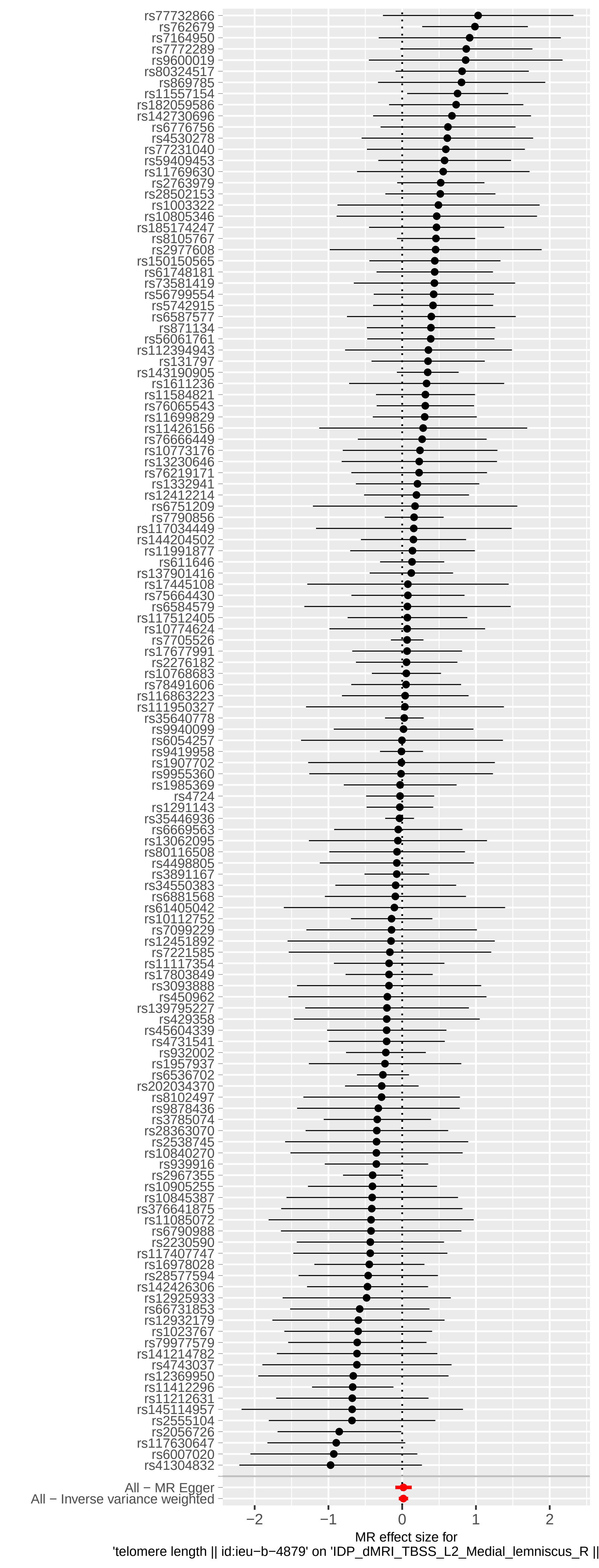
rs77732866
rs762679
rs7164950
rs7772289
rs9600019
rs80324517
rs869785
rs11557154
rs182059586
rs142730696
rs6776756
rs4530278
rs77231040
rs59409453
rs11769630
rs2763979
rs28502153
rs1003322
rs10805346
rs185174247
rs8105767
rs2977608
rs150150565
rs61748181
rs73581419
rs56799554
rs5742915
rs6587577
rs871134
rs56061761
rs112394943
rs131797
rs143190905
rs1611236
rs11584821
rs76065543
rs11699829
rs11426156
rs76666449
rs10773176
rs13230646
rs76219171
rs1332941
rs12412214
rs6751209
rs7790856
rs117034449
rs144204502
rs11991877
rs611646
rs137901416
rs17445108
rs75664430
rs6584579
rs117512405
rs10774624
rs7705526
rs17677991
rs2276182
rs10768683
rs78491606
rs116863223
rs111950327
rs35640778
rs9940099
rs6054257
rs9419958
rs1907702
rs9955360
rs1985369
rs4724
rs1291143
rs35446936
rs6669563
rs13062095
rs80116508
rs4498805
rs3891167
rs34550383
rs6881568
rs61405042
rs10112752
rs7099229
rs12451892
rs7221585
rs11117354
rs17803849
rs3093888
rs450962
rs139795227
rs429358
rs45604339
rs4731541
rs932002
rs1957937
rs6536702
rs202034370
rs8102497
rs9878436
rs3785074
rs28363070
rs2538745
rs10840270
rs939916
rs2967355
rs10905255
rs10845387
rs376641875
rs11085072
rs6790988
rs2230590
rs117407747
rs16978028
rs28577594
rs142426306
rs12925933
rs66731853
rs12932179
rs1023767
rs79977579
rs141214782
rs4743037
rs12369950
rs11412296
rs11212631
rs145114957
rs2555104
rs2056726
rs117630647
rs6007020
rs41304832
All − MR Egger
All − Inverse variance weighted
−2
−1
0
1
2
MR effect size for
'telomere length || id:ieu−b−4879' on 'IDP_dMRI_TBSS_L2_Medial_lemniscus_R ||
